# FAK regulates E-cadherin expression via p-Src^Y416^/p-ERK_1/2_/p-Stat3^Y705^ and PPAR_γ_/miR-125b/Stat3 signaling pathway in B16F10 melanoma cells

**DOI:** 10.18632/oncotarget.14687

**Published:** 2017-01-17

**Authors:** Guoshun Pei, Yan Lan, Dianhua Chen, Lina Ji, Zi-chun Hua

**Affiliations:** ^1^ The State Key Laboratory of Pharmaceutical Biotechnology, School of Life Sciences, Nanjing University, Nanjing, Jiangsu, 210046, China; ^2^ Changzhou High-Tech Research Institute of Nanjing University and Jiangsu Target Pharma Laboratories Inc., Changzhou, Jiangsu, 213164, China

**Keywords:** FAK, melanoma, miR-125b, E-Cadherin, migration

## Abstract

Focal adhesion kinase (FAK) is involved in tumor cell migration and metastasis. However, the underlying mechanism remains unclear. Here, we present a signaling pathway involved in the regulation of melanoma cell migration by FAK. We found that the interference of FAK expression suppressed B16F10 cell migration/metastasis, and altered the expressions of genes involved in melanoma migration/metastasis. The down-regulation of FAK inhibited the expression of p-Src^Y416^, p-ERK_1/2_, Stat3 and p-Stat3^Y705^, while promoted the expression of PPARγ, miR-125b and E-cadherin. Then we found that FAK inhibited E-cadherin expression via p-Src^Y416^/p-ERK_1/2_/ p-Stat3^Y705^ and PPARγ/miR-125b/Stat3 signaling pathway in B16F10 cell. Moreover, miR-125b inhibited B16F10 cell migration. Furthermore, we repeated the key data with human melanoma cell line A375. The results obtained from A375 cells fell in line with those from B16F10 cells. Using Oncomine database, we found that the mRNA levels of FAK, Src, ERK_1/2_ and Stat3 increased, while the mRNA levels of PPARγ, C21orf34 (miR-125b host gene) and E-cadherin decreased in human metastatic melanoma. The data from human breast cancer confirmed those from metastatic melanoma.

Taken together, our study suggests that down-regulation of FAK promotes E-cadherin expression via p-Src^Y416^/p-ERK_1/2_/p-Stat3^Y705^ and PPARγ/miR-125b/Stat3 signaling pathway. Our findings provide a novel explanation regarding how FAK promotes melanoma cell migration, suggesting that FAK might be a potential target for melanoma therapy.

## INTRODUCTION

Metastatic melanoma is one of the most challenging malignancies to cure, and is responsible for 60–80% of deaths from skin cancers [1, 2]. However, the mechanism underlying melanoma cell migration and metastasis is still poorly understood. Focal adhesion kinase (FAK) is a non-receptor protein tyrosine kinase that promotes cell adhesion, migration and invasion [3]. Recent studies showed that FAK was overexpressed in a variety of malignant tumors, and the level of the FAK was positively correlated with the malignant degree of melanoma [4]. The reduced expression of FAK blocked cell invasion, migration and metastasis in neuroblastoma [5]. Previously we reported that highly metastatic B16F10 cells had a higher FAK expression level than that of lowly metastatic B16 F1 cells [6].

E-cadherin is a single-pass transmembrane glycoprotein, which mediates cell-cell adhesions. Down-regulation of E-cadherin promoted cancer cell migration [7, 8]. Moreover, loss of E-cadherin also promoted invasive and metastatic behaviors in many epithelial tumors [9, 10]. The reduced FAK led to increased E-cadherin in breast tumor [11]. Recent study reported that small interfering RNA of Stat3 significantly increased E-cadherin expression, indicating that Stat3 negatively regulated E-cadherin expression [12].

In this study we explored the signaling pathway involved in the inhibition of E-cadherin by FAK. The role of FAK as a potential target for melanoma therapy was also discussed.

## RESULTS

### The knockdown of FAK suppressed B16F10 cell migration and tumor metastasis

**Figure 1:**
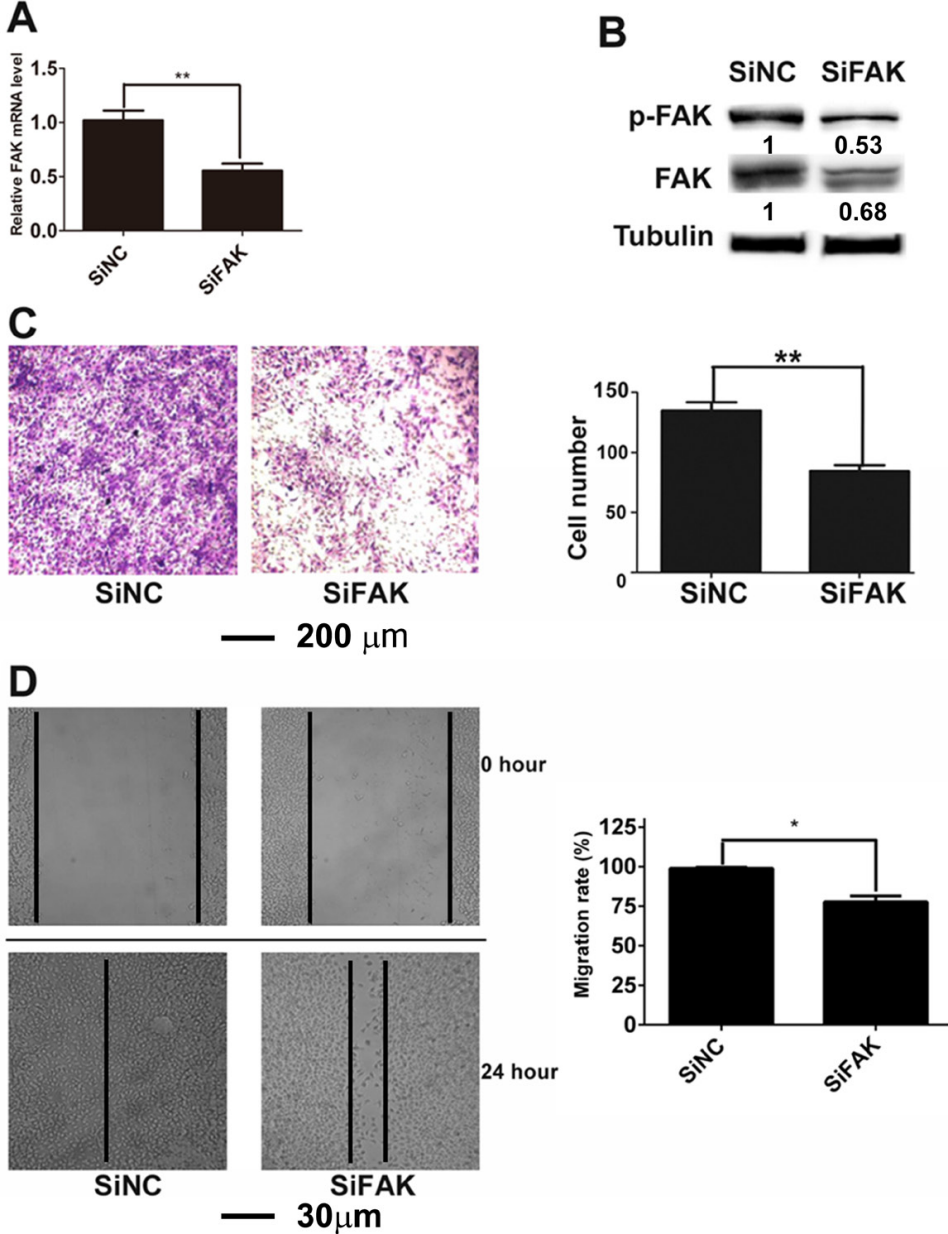
Down-regulation of FAK suppressed the migration of B16F10 cells (**A**) The mRNA level of FAK in SiNC and SiFAK cells. (**B**) The expression of FAK and p-FAK^Y397^ in SiNC and SiFAK cells. Normalized densitometry results are also presented. (**C**) Migration of SiNC and SiFAK cells analyzed by transwell assay. (**D**) Migration of SiNC and SiFAK cells analyzed by wound healing assay.

**Figure 2:**
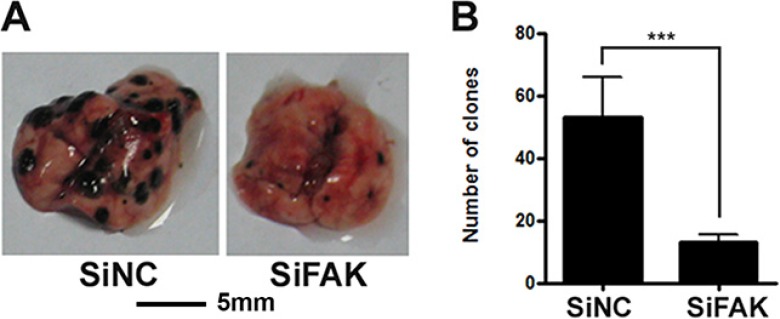
FAK promoted tumor metastasis (**A**) The tumor nodules formed on the mouse lungs that 17 days after mice were injected intravenously with SiNC and SiFAK cells. (**B**) Quantitative analysis of tumor nodules. The result was expressed as the mean ± SD.

We have reported that the expression level of FAK in highly metastatic B16F10 cells is higher than that in lowly metastatic B16F1 cells. To further explore the role of FAK in B16F10 cell migration/metastasis, we constructed SiFAK and SiNC cell lines. Compared with those in SiNC cells, the mRNA level of FAK (Figure 1A) and the protein levels of p-FAK and FAK (Figure 1B) all decreased markedly in SiFAK cells. The effect of FAK on cell migration was monitored by transwell assay (Figure 1C) and wound healing assay (Figure 1D). The down-regulation of FAK significantly suppressed B16F10 cell migration. The role of FAK in tumor metastasis *in vivo* was further investigated by intravenously injecting SiFAK or SiNC cells into C57BL/6J mice. As indicated by the decreased number of tumor nodules on the lung surface of mice injected with SiFAK cells, the down-regulation of FAK markedly suppressed tumor metastasis (Figure 2A and 2B).

### The expressions of genes involved in melanoma migration/metastasis were altered in SiFAK cells

**Figure 3:**
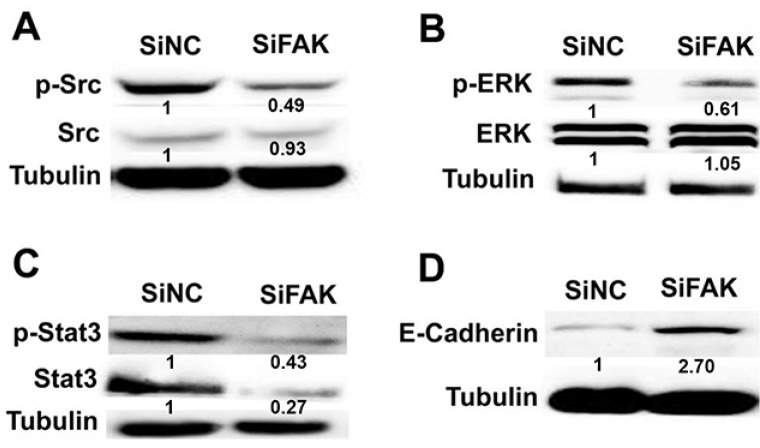
The effects of FAK on Src, p-Src^Y416^, ERK_1/2_, p-ERK_1/2_, Stat3, p-Stat3^Y705^ and E-Cadherin expression (**A**) The expression of Src and p-Src^Y416^, (**B**) ERK_1/2_ and p-ERK_1/2_, (**C**) Stat3 and p-Stat3^Y705^ and (**D**) E-cadherin expression in SiNC and SiFAK cells were examined by western blotting. Normalized densitometry results are also presented.

It was reported that the inhibition of FAK decreased invasion and metastatic potential of cancer [13]. The loss of FAK was associated with decreased ERK_1/2_ activity in mammary epithelial cells. Previous study indicated that the reduction in FAK expression also increased E-cadherin levels in tumor cells [14]. E-cadherin altered melanoma cell interactions and inhibited tumor cell invasion and metastasis. The loss of E-cadherin expression was common in melanoma [15–17]. To reveal the mechanism underlying the role of FAK in tumor migration/metastasis, we examined the effect of FAK knockdown on the levels of Src, p-Src^Y416^, ERK_1/2_, p-ERK_1/2_, Stat3, p-Stat3^Y705^ and E-cadherin by western blotting. The results showed that the stable interference of FAK expression in SiFAK cells decreased the levels of p-Src^Y416^ and p-ERK_1/2_ while did not affect those of total Src and ERK_1/2_ (Figure 3A and 3B). Compared with SiNC cells, the levels of Stat3 and p-Stat3^Y705^ decreased in SiFAK cells (Figure 3C). However, the interference of FAK significantly increased E-cadherin expression (Figure 3D).

### Down-regulation of FAK increased E-cadherin expression via p-Src^Y416^/p-ERK_1/2_/p-Stat3^Y705^ signaling pathway in B16F10 melanoma cell

**Figure 4:**
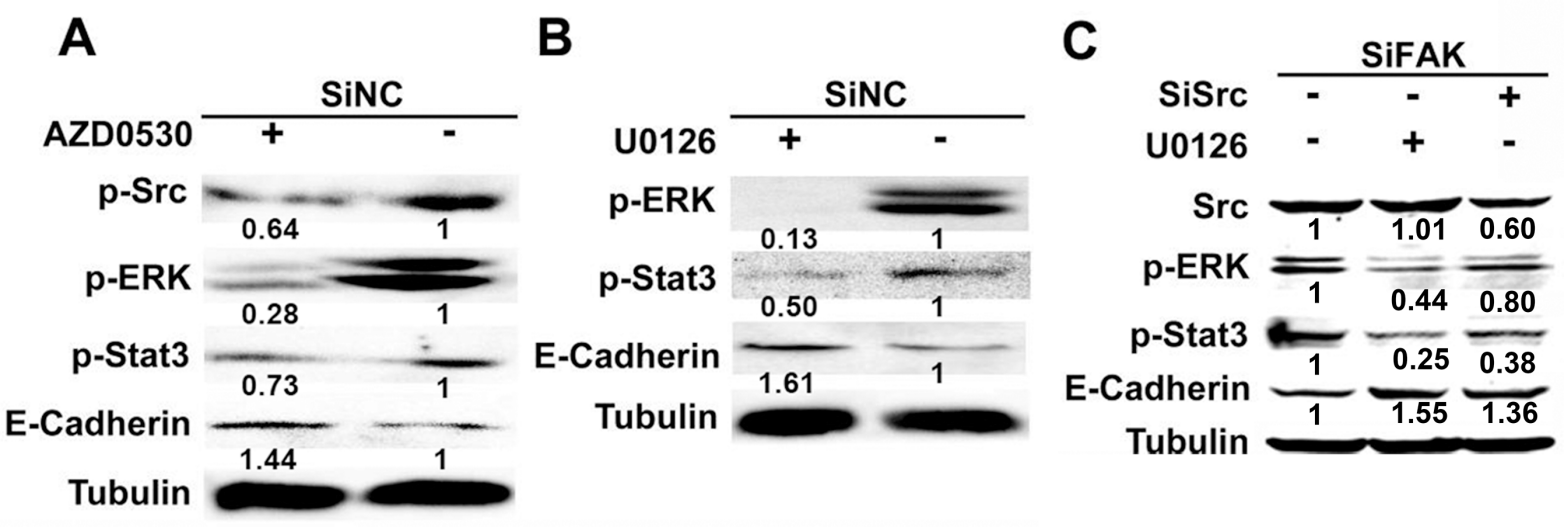
p-Src^Y416^/p-ERK_1/2_/p-Stat3^Y705^ signaling pathway was involved in FAK mediated E-cadherin expression (**A**) The expression of p-Src^Y416^, p-ERK_1/2_, p-Stat3^Y705^ and E-cadherin in SiNC cells incubated with or without Src inhibitor AZD0530 (5 μM) for 24 hours. (**B**) The expression of p-ERK_1/2_, p-Stat3^Y705^ and E-Cadherin in SiNC cells incubated with or without ERK_1/2_ inhibitor U0126 (10 μM) for 24 hours. (**C**) The expression of Src, p-ERK_1/2_, p-Stat3^Y705^ and E-cadherin in SiFAK cells incubated with U0126 (10 μM) or transfected with SiSrc. Normalized densitometry results are also presented.

The autophosphorylated FAK at Tyr^397^ (FAK^Y397^) can recruit and phosphorylate Src, followed by the phosphorylation of ERK_1/2_ by p-Src [18]. The inactivation of ERK_1/2_ decreased the phosphorylation of Stat3^Y705^ (p-Stat3^Y705^) in human gastric cancer cells [19]. In addition, there was a negative correlation between p-Stat3^Y705^ and E-cadherin expression in hepatocellular carcinoma [20]. Based on our previous data, we speculated that FAK might block the expression of E-cadherin via p-Src^Y416^/ p-ERK_1/2_/ p-Stat3^Y705^ signaling pathway in B16F10 cells. To verify this hypothesis, the SiNC and SiFAK cells were treated with Src inhibitor (AZD0530) or ERK_1/2_ inhibitor (U0126), and the protein levels of p-Src^Y416^, p-ERK_1/2_, p-Stat3^Y705^ and E-cadherin were explored by western blotting. When the phosphorylation of Src^Y416^ (p-Src^Y416^) was inhibited by AZD0530, the levels of p-ERK_1/2_ and p-Stat3^Y705^ dramatically decreased, while E-cadherin remarkably increased in SiNC cells (Figure 4A). When p-ERK_1/2_ was inhibited by U0126, p-Stat3^Y705^ markedly decreased while E-cadherin increased in SiNC cells (Figure 4B). Furthermore, the short interference RNA of Src (SiSrc) or U0126 were used to treat SiFAK cells, and their effects on Src, p-ERK_1/2_, p-Stat3^Y705^ and E-cadherin were explored by western blotting. SiSrc decreased the levels of p-ERK_1/2_ and p-Stat3^Y705^, and elevated the level of E-cadherin in SiFAK cells. Without any effect on Src expression, U0126 inhibited p-ERK_1/2_ and p-Stat3^Y705^, and promoted E-cadherin expression (Figure 4C). These data suggest that FAK inhibits E-cadherin expression via p-Src^Y416^/p-ERK_1/2_/p-Stat3^Y705^signaling pathway in B16F10 cells.

### FAK repressed E-cadherin expression via PPARγ/miR-125b/Stat3 signaling pathway

**Figure 5:**
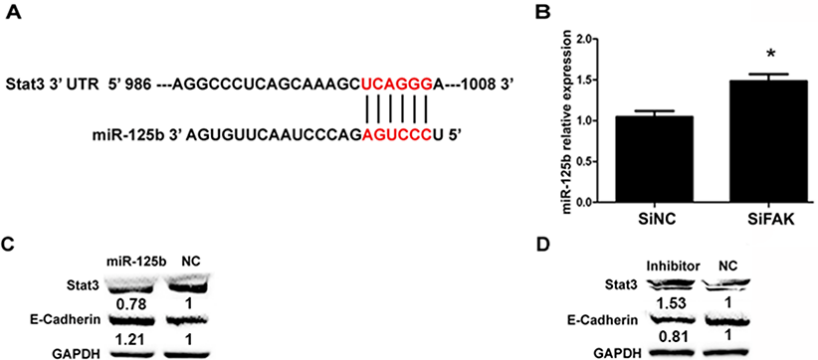
miR-125b promoted E-cadherin expression (**A**) The duplex between miR-125b and mouse stat3 3′ UTR predicted by mirbase (http://www.mirbase.org). (**B**) The expressions of miR-125b in SiNC and SiFAK cells were examined by QRT-PCR. The results were expressed as the mean ± SD. (**C**) The expressions of Stat3 and E-cadherin in SiFAK cells transfected with miR-125b mimic and NC. (**D**) The expressions of Stat3 and E-cadherin in SiFAK cells transfected with miR-125b inhibitor and NC. Normalized densitometry results are also presented.

As stated above, we found that FAK knockdown decreased Stat3 and p-Stat3^Y705^ (Figure 3C), and FAK activated p-Stat3^Y705^ via p-Src^Y416^/p-ERK_1/2_ signaling pathway. But it is unclear how FAK regulates Stat3 expression. Accumulating evidence revealed that miRNAs are dysregulated during melanoma progression [21, 22]. It is known that miRNAs bind to the 3′ untranslated region (UTR) of target mRNA to inhibit protein translation. Previous study has indicated that miR-125b expression is down-regulated in melanoma [23], and Stat3 is a downstream target of miR-125b [24]. Based on the analysis of publicly available databases, we also found that Stat3 was a potential target of miR-125b (Figure 5A). Our results from QRT-PCR showed that the level of miR-125b in SiFAK cells was significantly higher than that in SiNC cells, indicating miR-125b may participate in the regulation of Stat3 by FAK (Figure 5B). It is reported that knockdown of Stat3 significantly increased E-cadherin expression in colorectal cancer cells [25]. Therefore, FAK might inhibit E-cadherin expression via miR-125b/Stat3 signaling pathway in B16F10 cells. In our study, miR-125b mimic, miR-125b inhibitor and their negative controls were transfected into SiFAK cells, respectively. Compared to the negative control, miR-125b mimic decreased Stat3 while increased E-cadherin (Figure 5C). As expected, the effect of miR-125b inhibitor on the expressions of Stat3 and E-cadherin was contrary to that of miR-125b mimic (Figure 5D). Our results suggested that FAK inhibited E-cadherin expression via miR-125b/Stat3 signaling pathway in B16F10 melanoma cells.

**Figure 6:**
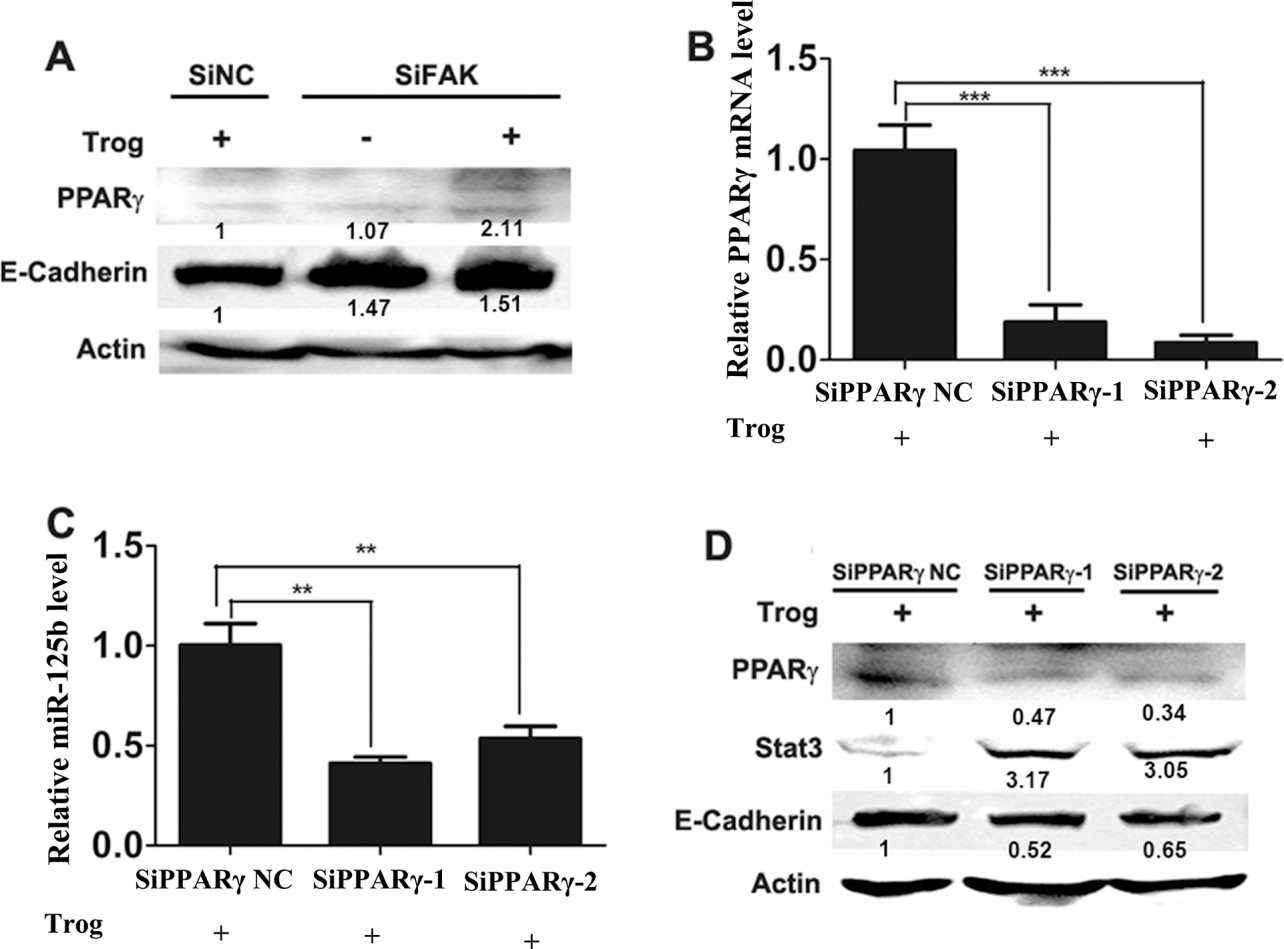
PPARγ promoted miR-125b expression (**A**) The expression of PPARγ and E-cadherin in SiNC and SiFAK cells that incubated with Trog (20 μM). (**B**) The expression of PPARγ mRNA in SiFAK cells that transfected with PPARγ interference RNA SiPPARγ-1, SiPPARγ-2 and treated with Trog (20 μM). (**C**) QRT-PCR examined the expression of miR-125b in SiFAK cells after transfected with PPARγ interference RNA SiPPARγ-1, SiPPARγ-2 and incubated with Trog (20 μM). (**D**) The expression of PPARγ, Stat3 and E-cadherin in SiFAK cells after transfected with PPARγ interference RNA SiPPARγ-1, SiPPARγ-2 and incubated with Trog (20 μM). Normalized densitometry results are also presented.

However, it is remains to be elucidated that how FAK inhibits the expression of miR-125b. It is known that PPARγ can promote the expression of miR-125b by directly binding to the responsive element in miR-125b gene promoter region [26]. In our study, we explored whether PPARγ participated in the regulation of miR-125b. Compared with those in SiNC cells, the protein levels of PPARγ and E-cadherin were higher in SiFAK cell after treatment with the activator of PPARγ (troglitamine, Trog) (Figure 6A). Then the interference RNAs of PPARγ (SiPPARγ-1 and SiPPARγ-2) and their negative control were transfected into SiFAK cells (Figure 6B). As a result, we found that miR125b was down-regulated after the knockdown of PPARγ (Figure 6C), suggesting that FAK inhibited miR-125b expression by repressing PPARγ expression. Meanwhile, the interference of PPARγ enhanced the protein level of Stat3 and decreased that of E-cadherin (Figure 6D). Taken together, FAK repressed E-cadherin expression via PPARγ/miR-125b/Stat3 signaling pathway.

### The miR-125b suppressed the migration of B16F10 cells

**Figure 7:**
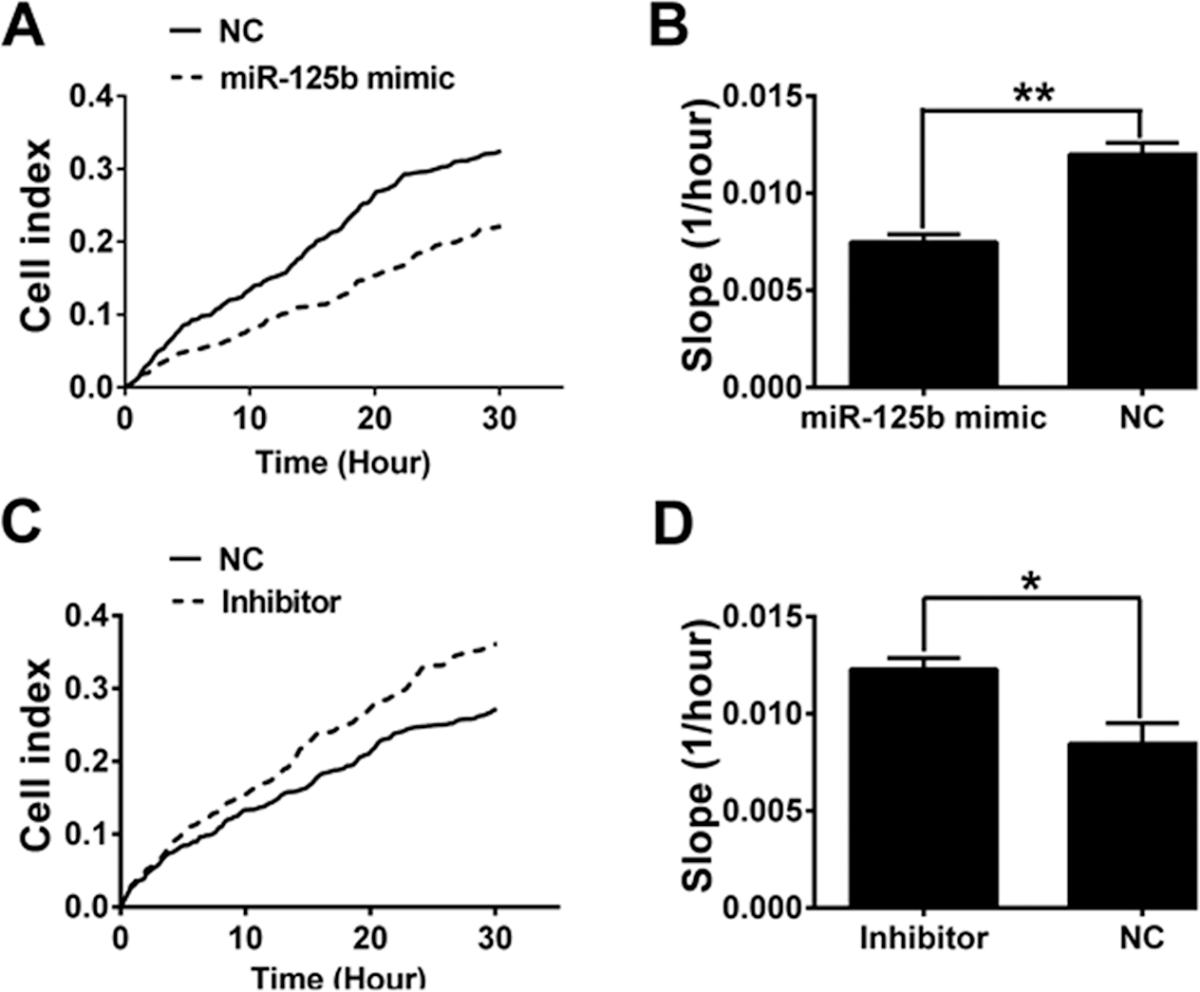
miR-125b inhibited B16F10 cell migration (**A**, **B**) The migrations of B16F10 cells transfected with miR-125b mimic and negative control were analyzed by RTCA. ** indicates *p* < 0.01. (**C**, **D**) The migrations of B16F10 cells transfected with miR-125b inhibitor and negative control were analyzed by RTCA. * indicates *p* < 0.05.

Recent study has suggested that miR-125b may function as a tumor suppressor in melanoma [27]. To test whether miR-125b can inhibit B16F10 cell migration, miR-125b mimic, miR-125b inhibitor and their negative control were transfected into B16F10 cells. The results from RTCA showed that miR-125b mimic significantly decreased the migration of B16F10 cells (Figure 7A and 7B), while miR-125b inhibitor remarkably increased the migration of B16F10 cells (Figure 7C and 7D). Our data indicated that miR-125b suppressed B16F10 cell migration.

### Dysregulated gene expressions of FAK, Src, ERK_1/2_, PPARγ, C21orf34, Stat3 and E-cadherin are associated with metastasis in melanoma patients

**Figure 8:**
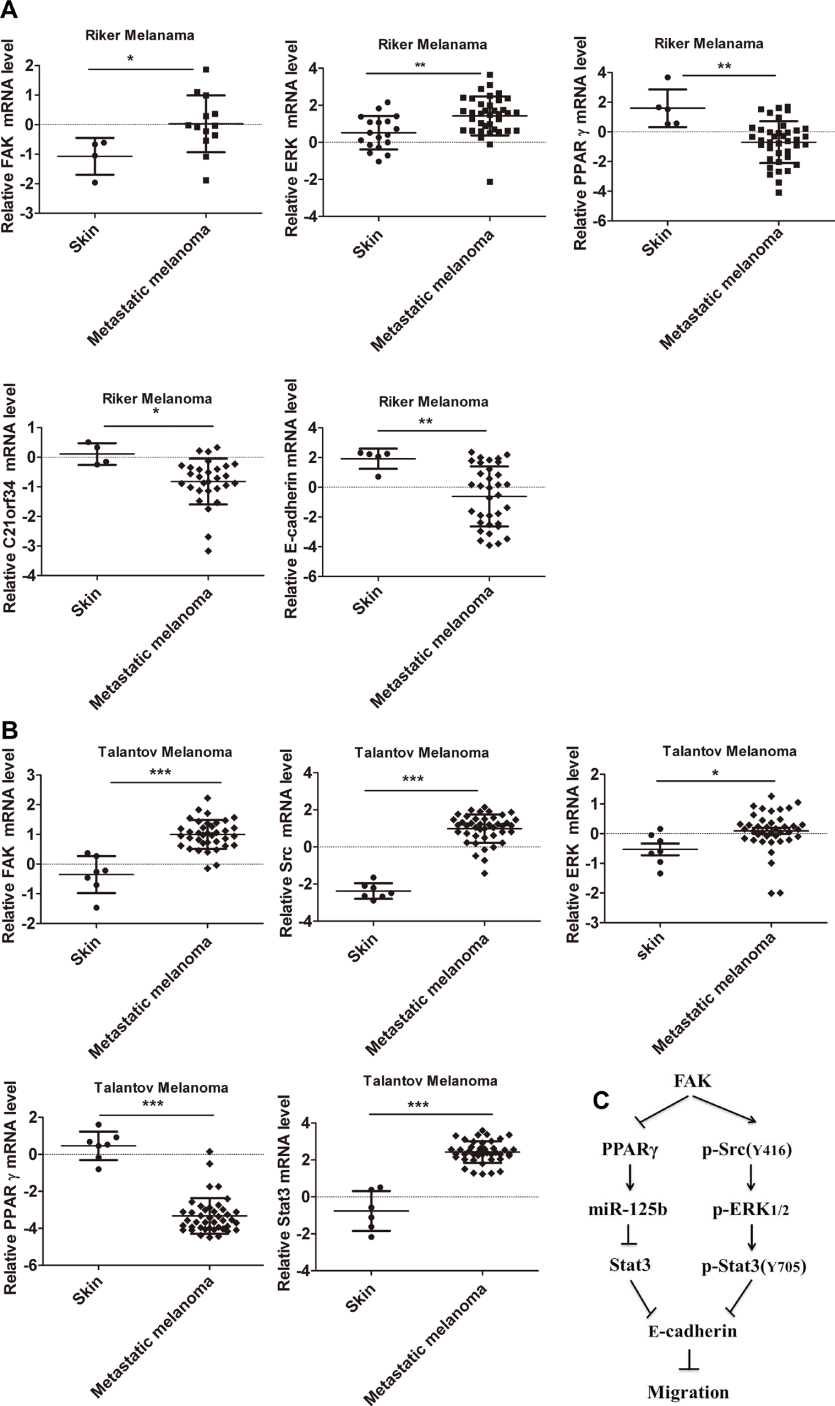
The mRNA levels of FAK, Src, ERK_1/2_, PPARγ, C21orf34, Stat3 and E-cadherin in human melanoma were obtained from Oncomine database (**A**) The mRNA levels of FAK, ERK_1/2_, PPARγ, C21orf34 and E-cadherin were analyzed in Riker melanoma dataset. (**B**) The mRNA levels of FAK, Src, ERK_1/2_, PPARγ and Stat3 were analyzed in Talantov melanoma dataset. *p < 0.05, **p < 0.01, ***p < 0.001. (**C**) Proposed model for the regulation of cell migration by FAK in melanoma cell.

In order to ascertain whether FAK, Src, ERK_1/2_, PPARγ, C21orf34 (miR-125b host gene), Stat3 and E-cadherin were differentially expressed in human melanoma tissues, we firstly analyzed their mRNA levels in melanoma cancer using Oncomine Cancer Microarray database (www.oncomine.org). As revealed by the data set of Riker melanoma, the expressions of FAK, ERK_1/2_ are up-regulated while the expressions of PPARγ, C21orf34 and E-cadherin are down-regulated in metastatic melanoma, as compared with normal controls (Figure 8A). As indicated by the data set of Talantov melanoma, compared with normal controls, the expressions of FAK, Src, ERK_1/2_ and Stat3 are up-regulated, while the expression of PPARγ is down-regulated in metastatic melanoma (Figure 8B). The results from Oncomine database are consistent with our findings from B16F10 cells, indicating our findings may be helpful for melanoma therapy.

We also analyzed the mRNA level of FAK, Src, ERK_1/2_, PPARγ, C21orf34, Stat3 and E-cadherin in human breast cancer using Oncomine Cancer Microarray database. Compared with normal controls, the expressions of FAK, Src, ERK_1/2_ and Stat3 are up-regulated, while the expressions of PPARγ, C21orf34 and E-cadherin are down-regulated in TCGA Breast Cancer (Supplementary Table 1) and Turashvili Breast Cancer (Supplementary Table 2). The results from breast cancer confirmed those from metastatic melanoma, indicating FAK regulates E-cadherin expression via p-Src^Y416^/ p-ERK_1/2_/p-Stat3^Y705^ and PPARγ/miR-125b/Stat3 signaling pathway (Figure 8C).

## DISCUSSION

In this study, we demonstrated that FAK knockdown inhibited B16F10 cell migration and metastasis, and further explored the underlying mechanism. Our data showed that down-regulation of FAK inhibited the phosphorylation of Src and ERK_1/2_. Besides, FAK knockdown suppressed the levels of Stat3 and p-Stat3^Y705^. The above results are consistent with the recent report that knockdown of FAK inhibits ERK_1/2_ activation in rat vascular SMCs [28], and reduction of p-ERK_1/2_ inhibits Stat3 activation in MCF-7 and MDA-MB-231 cells [29]. Furthermore, we revealed that FAK inhibited E-cadherin expression via P-Src^Y416^/p-ERK_1/2_/p-Stat3^Y705^ signaling pathway.

However, it remains to be elucidated how FAK signaling pathway inhibits Stat3 expression and whether Stat3 participates in the regulation of E-cadherin. Previous study has shown that miR-125b interacts with the 3′-UTR of the Stat3 mRNA to repress gene expression. Based on the publicly available database (mirbase, http://www.mirbase.org), Stat3 is found to be a potential target of miR-125b. Besides, recent study also reports that the expression of miR-125b is down-regulated in cutaneous malignant melanoma [30], and miR-125b acts as a tumor suppressor in hepatic tumor and cutaneous malignant melanoma [31, 32]. Therefore, we further investigated the role of miR-125b in B16F10 cell. We found that FAK knockdown promoted the expression of miR-125b. The miR-125b mimic inhibited Stat3 expression while promoted E-cadherin expression. Based on further study, we found that FAK repressed miR-125b expression via down-regulation of PPARγ. In addition, we found that miR-125b could inhibit B16F10 cell migration.

All the above data obtained from mouse cell line B16F10 suggest that FAK regulates E-cadherin expression via p-Src^Y416^/p-ERK_1/2_/p-Stat3^Y705^ and PPARγ/miR-125b/Stat3 signaling pathway. We also repeated the key experiments in human melanoma cell line A375. The interference RNA of FAK and its negative control were transfected into A375 cells, and the levels of several proteins were examined by western blotting. When the expression of FAK was blocked, the protein levels of p-Src^Y416^, p-ERK_1/2_, Stat3 and p-Stat3^Y705^ were down-regulated, while the protein levels of PPARγ and E-cadherin were up-regulated (Supplementary Figure 1A). Then the interference RNA of Src (Supplementary Figure 1B), U0126 (Supplementary Figure 1C) and the interference RNA of PPARγ (Supplementary Figure 1C) were used to treat A375 cells, and the protein levels of Src, p-ERK_1/2_, PPARγ, Stat3, p-Stat3^Y705^ and E-cadherin were examined. The results from A375 cells fall in line with those obtained from B16F10 cells, indicating the two cell lines share a common pathway underlying the repression of E-cadherin expression by FAK.

Mouse melanoma cell lines B16F10 and B16F1 were obtained from their parent cell line B16F0 by clonal selection of metastatic tumor [6]. Consistent with the reference, we found that the expression level of FAK in highly metastatic B16F10 cells is much higher than that in lowly metastatic B16F1 cells (Supplementary Figure 2). Using B16F10 and B16F1 cell lines, we investigated the effect of up-regulated FAK on genes involved in melanoma migration/ metastasis. Compared with those in B16F10 cells, the protein levels of p-Src^Y416^, p-ERK_1/2_, Stat3 and p-Stat3^Y705^ decreased, while the protein levels of PPARγ and E-cadherin increased in B16F1cells (Supplementary Figure 2). The above results fell in line with those obtained from SiFAK and SiNC cells, suggesting FAK/ p-Src^Y416^/ p-ERK_1/2_/p-Stat3^Y705^/E-cadherin and FAK/PPARγ/miR-125b/Stat3/E-cadherin signaling pathway also works when FAK expression is up-regulated.

As indicated by data from Oncomine database, compared with normal controls, the expressions of FAK, Src, ERK_1/2_, Stat3 mRNA increased, while the expressions of PPARγ, C21orf34 and E-cadherin decreased in human melanoma cancer and breast cancer. In conclusion, our data indicated that FAK inhibited the expression of E-cadherin via p-Src^Y416^/p-ERK_1/2_/ p-Stat3^Y705^ and PPARγ/miR-125b/Stat3 signaling pathway in both melanoma cells and human tumors. Our study suggests that FAK may be a potential target for melanoma therapy.

## MATERIALS AND METHODS

### Biochemicals and antibodies

AZD0530, U0126 and blasticidin were obtained from Sigma-Aldrich, Cell Signaling and Invitrogen, respectively. Primary antibodies against FAK, phosphorylated FAK (p-FAK^Y397^) and E-cadherin were purchased from BD Biosciences. Primary antibodies against Src, phosphorylated Src (p-Src^Y416^), Stat3, phosphorylated Stat3 (p-Stat3^Y705^), ERK_1/2_, phosphorylated ERK_1/2_ (p-ERK_1/2_) and PPARγ were obtained from Cell Signaling. Primary antibodies against tubulin, actin and GAPDH were obtained from Santa Cruz.

### Cell cultures

SiFAK cell line (stable interference of FAK in B16F10 cells) and SiNC cell line (negative control for SiFAK) were constructed by stable transfection of plasmid pcDNA6.2-GW/EmGFP-miR harboring FAK small interfering RNA or siFAK scramble siRNA into B16F10 cells, respectively [33]. Cells were cultured at 37°C and 5% CO_2_ in Dulbecco's modified Eagle's medium (DMEM) supplemented with 10% fetal bovine serum (Life Technologies, USA) and 3 mg·L^−1^ blasticidin.

### Migration assay

Cell migration was monitored by transwell assay, real-time cell analyzer (RTCA) and wound healing assays. For transwell assay, 1 × 10^5^ cells per insert (8 μm pore size) were incubated in serum-free DMEM for 24 hours. Then cells inside the inserts were removed with cotton swab, while cells at underside of the inserts were fixed and stained. Taken photographs of five fields and counted the total cells, the average number of cells per field was calculated.

The ×CELLigence real-time cell analyzer (RTCA) DP system was used to monitor cell adhesion. After equilibration of the CIM-Plate-16 plates for 1 hour in a humidified atmosphere at 37°C, 2.0 × 10^4^ cells/well in serum-free medium were transferred into CIM-Plate-16 with 3 replicates. The impedance was monitored continually for 30 hours according to the manufacturer's instructions, and data were shown as a cell index. For the wound healing assay, cells were seeded at an initial density of 2 × 10^5^ cells/well, and cultured in DMEM supplemented with 10 % fetal bovine serum overnight. Then cells were cultured in serum-free DMEM for 24 hours. A micro pipette tip was used to create a wound in the monolayer of cells. Wound closures were observed by phase-contrast microscopy, and digital images were taken at the interval time of 0 hour and 24 hour.

### Tail vein metastasis

Female C57BL/6J mice (6–8 weeks old) were purchased from Comparative Medicine Center of Yangzhou University (Yangzhou, China). C57BL/6J mice were injected intravenously with SiFAK or SiNC cells (5 × 10^5^) via the tail. After 17 days, the mice were euthanized, and their lungs were harvested and photographed. The numbers of tumor nodules on the surface of lungs were counted under macroscope.

### Total RNA isolation and quantitative real time PCR (QRT-PCR)

Total RNA was extracted from SiNC and SiFAK cell line by the TRIzol reagent (Invitrogen, USA). RNA quantity and purity were determined by Bio-photometer (Eppendorf, Germany). First-strand cDNA was synthesized with 1.5 ug of total RNA using PrimeScript RT reagent kit (Takara, Japan). QRT-PCR was performed by using FastStart Universal SYBR Green Master [Rox] (Roche, Swiss). The gene-specific primers were synthesized by Nanjing Genscript (Nanjing, China). U6 was used to normalize the expression data of miR-125b.

U6-RT: 5′-CTCAACTGGTGTCGTGGAGTCGGC AATTCAGTTGAGAAAAATATGGAACGCT-3′ U6-F: 5′-CTGGTAGGGTGCTCGCTTCGGCAG-3′ U6- R: 5′- CAACTGGTGTCGTGGAGTCGGC-3′ miR-125b- RT: 5′-CTCAACTGGTGTCGTGGAGTCGGCAATTCAGTT GAGTACAA-3′ miR-125b -F:5′-CGCGCTCCCTGAGA CCCTAAC-3′ miR-125b- R: 5′-TGGTGTCGTGGAG TCG-3′ FAK-F: 5′-AAAGCAGTAGTGAGCCAACAA-3′ FAK-R: 5′-CTGAGGCGAAATCCATAGC-3 PPARγ-F: 5′- ATCTTAACTGCCGGATCCAC -3′ PPARγ-R: 5′- GA TGGCATTGTGAGACATCC -3′ GAPDH-F: 5′-TGAAGC AGGCATCTGAGGG-3′ GAPDH-R: 5′-CGAAGGTGGA AGAGTGGGAG-3′.

### RNA transfection

miR-125b mimic, miR-125b mimic negative control (NC), miR-125b inhibitor, miR-125b inhibitor NC, interference RNA of Src (SiSrc) and interference RNA of PPARγ (SiPPARγ) oligos were synthesized by GenePharma (Shanghai, China). SiPPARγ-1-F: 5′-GCGAUCUUGACAGGAAAGATT-3′ SiPPARγ-1-R: 5′-UCUUUCCUGUCAAGAUCGCTT-3′ SiPPARγ-2-F: 5′-GACAGUGACUUGGCUAUAUTT-3′ SiPPARγ-2-R: 5′-AUAUAGCCAAGUCACUGUCTT-3′ SiSrc-F: 5′-CU GUAUCCGACUUCGACAATT -3′ SiSrc-R: 5′-UUGU CGAAGUCGGAUACAGTT-3′ miR-125b mimic-F: 5′-UCCCUGAGACCCUAACUUGUGA-3′ miR-125b mimic-R: 5′-ACAAGUUAGGGUCUCAGGGAUU-3′ NC for SiPPARγ, SiSrc and miR-125b mimic NC-F: 5′-U UCUCCGAACGUGUCACGUTT-3′ NC-R: 5′-ACGUG ACACGUUCGGAGAATT-3′ miR-125b inhibitor: 5′-UCA CAAGUUAGGGUCUCAGGGA-3′ miR-125b inhibitor- NC: 5′-CAGUACUUUUGUGUAGUACAA-3′ Cells were transfected with RNA according to the protocol of Lipofectamine 2000 (Invitrogen, USA), and cultured for 48 hours before harvest.

### Western blotting

Cells were lyzed and total proteins were fractionated using SDS-PAGE and transferred onto nitrocellulose membrane. The membrane was blocked with 5% non-fat dried milk in 1 × PBST buffer and then incubated with appropriate primary antibodies for one hour. Horseradish peroxidase-conjugated anti-mouse or anti-rabbit IgG was used as the secondary antibody, the protein bands were detected using the enhanced chemiluminesence detection system (Tanon, Shanghai, China). The density of different protein bands was analyzed by Chemianalysis software, and the value of density analysis was labeled under each protein band.

### Statistical analysis

Three replicates were performed for the experiments. The data are presented as the mean ± S.D, and analyzed using Student's *t* test. Statistical significance was defined as follows: *p < 0.05; **p < 0.01; ***p < 0.001.

### Abbreviations

FAK: Focal adhesion kinase; GAPDH: glyceraldehyde-3-phosphate dehydrogenase; NC: normal control; PBST: phosphate buffer saline with Tween; QRT-PCR: quantitative real time PCR; 3′-UTR: 3′-untranslated regions; Trog: troglitamine; SiFAK cell line: stable interference of FAK in B16F10 cells; SiNC cell line: negative control for SiFAK cell line; RTCA: real-time cell analyzer.

### Consent for publication

Not applicable.

### Ethics approval and consent to participate

All animal use protocols were approved by the Animal Care and Use Committee of the School of Life Sciences of Nanjing University.

## SUPPLEMENTARY MATERIALS


