# Supplementary material for: FAK regulates E-cadherin expression via p-SrcY416/p-ERK1/2/p-Stat3Y705 and PPARγ/miR-125b/Stat3 signaling pathway in B16F10 melanoma cells
Source: Oncotarget. 2017 Jan 17;8(8):13898–908. doi: 10.18632/oncotarget.14687 (PMC5355148; doi:10.18632/oncotarget.14687)
Supplement: Supplementary file 1 [file oncotarget-08-13898-s001.pdf]

FAK regulates E-cadherin expression via p-Src^{Y416}/p-ERK$_{1/2}$/p-Stat3^{Y705} and PPARγ/miR-125b/Stat3 signaling pathway in B16F10 melanoma cells

Supplementary Materials

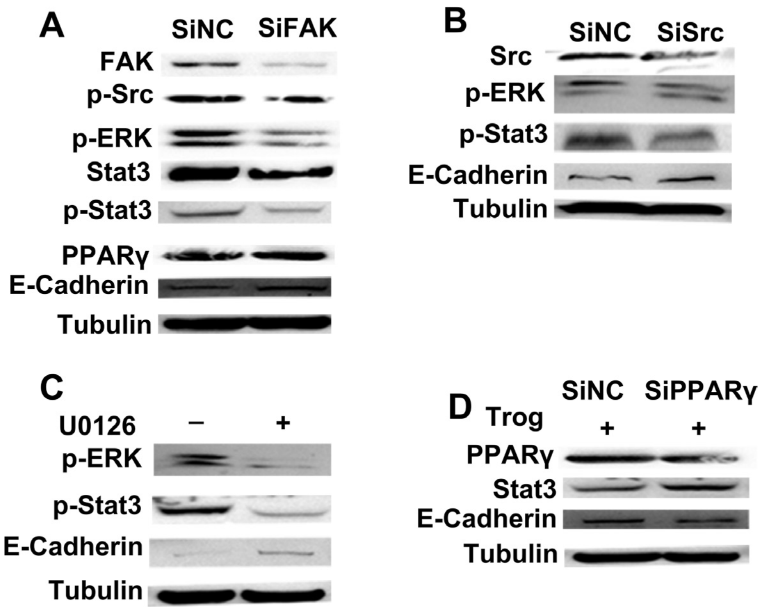

Supplementary Figure 1: FAK regulates E-cadherin expression via p-Src^{Y416}/p-ERK$_{1/2}$/p-Stat3^{Y705} and PPARγ/miR-125b/Stat3 signaling pathway in human melanoma cell line A375. (**A**) The expressions of FAK, p-Src^{Y416}, p-ERK$_{1/2}$, PPARγ, Stat3, p-Stat3^{Y705} and E-Cadherin in A375 cells transfected with SiNC or SiFAK were examined by western blotting. (**B**) The expressions of Src, p-ERK$_{1/2}$, p-Stat3^{Y705} and E-cadherin in A375 cells transfected with SiNC or SiSrc were examined by western blotting. (**C**) The expressions of p-ERK$_{1/2}$, p-Stat3^{Y705} and E-cadherin in A375 cells treated with U0126 were examined by western blotting. (**D**) The expressions of PPARγ, Stat3 and E-cadherin in A375 cells transfected with SiNC or SiPPARγ were examined by western blotting.

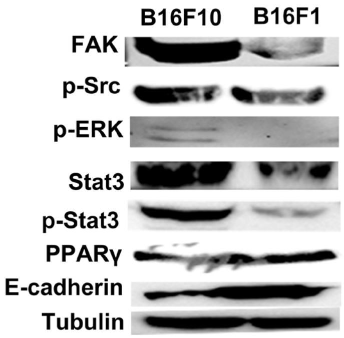

Supplementary Figure 2: The expressions of FAK, p-Src^{Y416}, p-ERK$_{1/2}$, Stat3, p-Stat3^{Y70}5, PPARγ and E-Cadherin in B16F10 and B16F1 cells were examined by western blotting.

Supplementary Table 1: The mRNA levels of FAK, Src, ERK, PPARγ, C21orf34, Stat3 and E-Cadherin in TCGA Breast Cancer

Gene	Fold change (Invasive Lobular Carcinoma vs Normal)
FAK	1.28
Src	1.26
ERK	1.41
PPARγ	−4.69
C21orf34	−2.51
Stat3	1.56
E-cadherin	−3.68

The fold change (Invasive Lobular Carcinoma vs Normal) is a relative value. The value > 0 represents the expression of gene is up-regulated in tumor, compared with that in normal control, and *vice versa*.

Supplementary Table 2: The mRNA levels of FAK, Src, ERK, PPARγ, C21orf34, Stat3 and E-Cadherin in Turashvili Breast Cancer

Gene	Fold change (Invasive Ductal Breast Cancer vs Normal)
FAK	1.91
Src	1.38
ERK	1.88
PPARγ	−1.26
C21orf34	−1.19
Stat3	1.10
E-cadherin	−1.38

The fold change (Invasive Ductal Breast Cancer vs Normal) is a relative value. The value > 0 represents the expression of gene is up-regulated in tumor, compared with that in normal control, and *vice versa*.
